# Multidimensional Analysis of Physical, Psychosocial, and Cognitive Impairment in People with Chronic Neck Pain

**DOI:** 10.3390/medicina62050956

**Published:** 2026-05-14

**Authors:** Zeynep Guven, Ezgi Yetim Arsava, Ahmet Ilkay Isikay, Ayse Akyay, Erdem Karabulut, Songul Atasavun Uysal

**Affiliations:** 1 Faculty of Physical Therapy and Rehabilitation, Hacettepe University, 06100 Ankara, Turkey; songula@hacettepe.edu.tr; 2 Department of Neurology, Faculty of Medicine, Hacettepe University, 06230 Ankara, Turkey; ezgiyetim@hacettepe.edu.tr (E.Y.A.); akyayayse2@gmail.com (A.A.); 3 Department of Neurosurgery, Faculty of Medicine, Hacettepe University, 06230 Ankara, Turkey; ilkayisikay@gmail.com; 4 Department of Biostatistics, Faculty of Medicine, Hacettepe University, 06230 Ankara, Turkey; ekarabul@hacettepe.edu.tr

**Keywords:** neck pain, mental health, kinesiophobia, muscle strength, physical capacity, neuropsychological tests, chronic pain

## Abstract

*Background and Objectives*: Chronic neck pain is a prevalent condition linked to functional disability and maladaptive pain-related behaviors. Although the physical and psychosocial impairments have been extensively studied in the literature, the factors related to cognitive impairments are not fully understood. This study aimed to examine the physical, psychosocial, and cognitive impairments associated with chronic neck pain and examine the variables associated with cognitive performance in this population. *Materials and Methods*: This cross-sectional study included 87 individuals with chronic neck pain. Pain sensitivity, neck disability, physical capacity, cervical muscle strength, pain catastrophizing, kinesiophobia, and self-reported central sensitization were assessed in all participants. Within the scope of the neuropsychological assessment, global cognition, processing speed, attention, and executive functioning were evaluated. The relationship between physical, psychosocial, and cognitive impairments was assessed using Spearman correlation. Additionally, a multiple linear regression analysis was performed to assess the extent to which the independent variables were associated with cognitive performance. *Results*: Global cognition was moderately correlated with pain intensity (r = −0.427, *p* < 0.001) and pain catastrophizing (r = −0.414, *p* < 0.001). Difficulties in response inhibition were moderately related to age (r = 0.440, *p* < 0.001). Cervical muscle strength (r = 0.384–0.233) and physical capacity (r = 0.332) were also weakly correlated with global cognition (*p* < 0.05). Age exhibited the strongest relationship with global cognition (β = −0.332, *p* < 0.001), followed by pain intensity (β = −0.291, *p* = 0.004) and pain sensitivity (β = 0.253, *p* = 0.011), with an explained variance of 30.8%. Additionally, age showed the strongest association with difficulties in processing speed (β = 0.448, *p* < 0.001), followed by kinesiophobia (β = 0.325, *p* = 0.001) and neck disability (β = 0.262, *p* = 0.030), with an explained variance of 34.2%. *Conclusions*: These findings suggest that increased nociceptive sensitization and deficits in physical health may be associated with maladaptive pain-related coping strategies in individuals with chronic neck pain. Furthermore, cognitive impairments in these individuals may reflect a multidimensional association of demographic, physical, psychosocial, and sensorial mechanisms.

## 1. Introduction

Chronic neck pain is a common and often disabling condition that leads to pain-related disability, functional limitations, and a substantial ongoing personal and healthcare burden worldwide [1,2]. According to the study conducted by Global Burden of Diseases 2021 neck pain collaborators, 203 million people worldwide are diagnosed with neck pain, and years lived with disability reached 20.2 million [1]. In addition, nearly 70% of the global population experiences neck pain at least once in their lifetime, and 50% to 85% of these cases become recurrent or chronic within five years [3,4,5].

Several physical, clinical, sociodemographic, and psychological risk factors associated with chronic neck pain have been identified, including older age, female gender, unsuitable working conditions, stress, depression, and a history of musculoskeletal disorders [2,6]. Despite the increasing prevalence of neck pain and its known effects on psychosocial and physical health, some potential risk factors have not yet been clearly identified [1]. Considering that chronic neck pain may negatively affect individuals’ quality of life, psychological health, and occupational performance, understanding the risk factors is important for developing comprehensive prevention and management strategies for individuals with chronic neck pain [2,7,8].

Recent studies have comprehensively examined the effects of chronic neck pain on physical health and the cervical motor system. These studies have indicated a decrease in cervical range of motion, neck muscle strength, and endurance in individuals with neck pain [9,10,11,12]. These changes may affect the kinematics of neck movements, resulting in delayed muscle contraction response, earlier muscle fatigue, and increased muscle co-contractions and superficial muscle activation [11,13,14,15]. Furthermore, it has been shown in patients with whiplash-associated neck pain that the number of active myofascial trigger points may be related to perceived pain and the spontaneous pain area [16]. The increased muscle activation and existence of active myofascial trigger points may be associated with increased pain perception and neck disability levels, in addition to physical disabilities, in individuals with chronic neck pain [15,16,17].

In addition to the physical and motor system effects described above, individuals with chronic neck pain may also experience abnormal pain processing related to the sensory system [18]. The role of peripheral and central sensitization in different chronic pain conditions is subject to increasing interest, and the number of studies in the field addressing the management of pain through the mitigation of peripheral and central sensitization is steadily increasing [19,20,21]. Previous studies have reported that individuals with chronic neck pain may experience increased pain sensitivity due to the increased peripheral and central sensitization [22,23], and an increase in pain sensitivity may be associated with increased pain perception and functional disability in this population [24].

Individuals who experience abnormal pain processing tendencies are also likely to perceive pain as a threat, feel helpless in the presence of pain, and have difficulty inhibiting pain-related thoughts and activity avoidance [25,26,27]. Research has also shown increased central sensitization, pain catastrophizing behavior, and kinesiophobia in individuals with neck pain [28,29,30]. The kinesiophobia, central sensitization, and pain catastrophizing observed in this population may be considered indicators of pain-related fear-avoidance behavior, and increased levels of pain-related maladaptive behavior may contribute to increased disability, activity avoidance, and pain intensity [26,31].

Alongside research on the physical and psychosocial health of individuals with chronic neck pain, the potential impact of chronic pain on cognitive performance has increasingly been recognized as an important topic in the literature in recent years. Studies evaluating cognitive performance in different chronic pain conditions have indicated that people with chronic pain have difficulties, particularly regarding attention, memory, executive functions, and information processing speed, compared to healthy individuals. The reasons for this include factors such as pain duration, central sensitization, stress, and increased serum cortisol concentration [32,33,34]. Regardless of the initial causes of chronic pain, the presence of pain is associated with changes occurring in the central nervous system, and this leads to a decrease in cognitive performance [32,35]. Studies conducted on the impairment of cognitive performance in individuals with chronic neck pain also confirm this information, reporting that these individuals have difficulties in attention, memory, reaction time, and cognitive flexibility compared to healthy individuals [22,36].

Although current findings provide important insights into the relationship between pain and reduced cognitive function in this population, further clinical studies are required to confirm and better characterize this impairment. Previous studies have demonstrated that decreased cognitive performance is associated with increased pain intensity, central sensitization, neck disability, and poorer quality of life in people with chronic neck pain [22,37]. However, the relationship between pain-related maladaptive coping strategies and cognitive performance remains insufficiently investigated in this population. Additionally, to the best of our knowledge, evidence regarding the relationship between physical capacity, neuromuscular capacity and cognitive function remains limited, particularly in this population. Findings from previous studies are consistent with the biopsychosocial model, suggesting that impairments related to chronic pain are not solely associated with increased peripheral nociception, and that psychosocial and cognitive impairments should also be considered in the clinical picture [38,39]. There are a limited number of studies that address cognitive, physical, and psychosocial domains together in people with chronic neck pain. Furthermore, considering that the biopsychosocial model describes pain and pain-related disability as a multidimensional, dynamic interaction among physiological and psychosocial parameters that collectively contribute to the onset of chronic pain [40], examining the interactions between physical, psychosocial, and cognitive parameters together—rather than treating them as separate constructs—could provide unique insights into the clinical picture of chronic neck pain that isolated analyses cannot offer. In light of the gaps in the literature, this study aims to examine the physical, psychosocial, and cognitive impairments associated with chronic pain in individuals with chronic neck pain and investigate the associations between physical, neuromuscular, and psychosocial impairments and cognitive performance in this population, thereby contributing to the existing literature.

## 2. Materials and Methods

### 2.1. Study Design and Participants

The protocol of this cross-sectional study was approved by Hacettepe University Clinical Research Ethics Committee (Approval Decision Number: 2024/03-01, Research Number: KA-23031, Date: 20 February 2024). The study was conducted in accordance with the Declaration of Helsinki. All participants gave written informed consent for participation in the study.

This study was conducted between May 2025 and January 2026 at Hacettepe University Faculty of Physical Therapy and Rehabilitation. The participant inclusion criteria were as follows: aged between 20 and 65 years, complaining of neck pain for at least 3 months, scoring 24 points or above on the Standardized Mini-Mental State Test, and being at least a primary school graduate. The participants were excluded if they had a psychiatric diagnosis, had a neurological, metabolic, cardiovascular, and/or inflammatory systemic disease, had participated in a rehabilitation program for neck pain within the last 6 months, had a history of neck or shoulder surgery, or were pregnant, or if less than one year had passed since their last pregnancy. In addition, the participants were informed not to have used analgesics within the last 24 h.

All participants were diagnosed by a neurosurgeon (A.I.I.), and the participants’ eligibility for inclusion was confirmed by this physician. Neuropsychological assessments were administered by a clinical psychologist (A.A.), and the results were interpreted by a neurologist (E.Y.A). Physical assessments were carried out by a physiotherapist (Z.G). Neuropsychological assessments lasted approximately 30 min, while the remaining assessments required about one hour. To minimize participant fatigue, neuropsychological and physical assessments were conducted on separate days. All assessments were performed in examination rooms that were quiet, well-ventilated, and adequately illuminated to reduce potential confounding factors.

After individuals who met the inclusion criteria agreed to participate in the study, informed consent forms were obtained from them, and they were informed about the assessments to be conducted. On the first day of the assessments, after the demographic data collection and physical assessments, psychosocial questionnaires were administered to the participants. To minimize the potential effects of fatigue during physical assessments, muscle strength, physical capacity, and pressure pain threshold measurements were administered in a randomized order. To ensure pain and fatigue were kept under control during the tests, a 5 min rest period was provided between measurements, and the next measurement was initiated only after the patients indicated they were ready. After the physical assessments were completed, participants were asked to complete the questionnaires for neck disability, kinesiophobia, pain catastrophizing, and central sensitization under the supervision of a physical therapist. Neuropsychological assessments were administered on the second day of the assessments. In the neuropsychological assessments, the order of administration was randomized to minimize the effects of learning and fatigue, and 5 min rest periods were implemented between assessments.

### 2.2. Outcome Measures

#### 2.2.1. Demographic Data Collection

Demographic information was obtained directly from the participants. A demographic data collection form was used to record the demographic information, including the age, gender, height, weight, years of education, and occupation of participants. After obtaining occupation information, participants were scored according to the job scores developed by Vemuri et al. [41]. According to this scoring system, level 1 includes people without an occupation; level 2 includes service, transportation, private household, and material-moving occupations; level 3 includes sales, farming, administrative support, machine operating, and protective service occupations; level 4 includes technicians and precision manufacturing workers; level 5 includes executive, managerial, and administrative occupations; and level 6 includes professional specialty occupations [41].

#### 2.2.2. Edinburgh Hand Preference Questionnaire (EDHP)

The EDHP is one of the most frequently used questionnaires for determining the dominant hand [42,43]. The scale is scored between −100 (for left-handed) and +100 (for right-handed). Individuals scoring above +40 are classified as right-handed; those scoring between +40 and −40 (inclusive) are classified as ambidextrous (actively using both hands); and those scoring below −40 are classified as left-handed [42].

#### 2.2.3. Pain Intensity

Numerical scales are frequently used as one-dimensional pain intensity scales because they are easy to use and effective. When administering them, volunteers are asked to choose the number that best describes their pain intensity. Zero indicates no pain, while 10 represents the worst pain imaginable [44].

#### 2.2.4. Neck Disability Index (NDI)

Neck disability was rated using the NDI [12]. This questionnaire is commonly used, and its reliability and validity have been demonstrated [45,46]. The scale assesses subjective symptoms and activities of daily living. Items are scored between 0 (no limitation) and 5 (maximum limitation). Higher scores on this scale represent higher levels of pain-related disability [45].

#### 2.2.5. Pressure Pain Thresholds (PPTs)

PPTs for local symptomatic and distal asymptomatic regions were measured to assess pain sensitivity. This has been reported to be a reliable method that is widely used in both clinical and research settings [29,47]. PPTs were evaluated on the more painful side. In cases where patients experienced the same amount of neck pain on both sides, PPTs were tested on the dominant hand side. A digital pressure algometer (Wagner FPIX™ Digital Algometer, Greenwich, CT, USA) was used to evaluate PPTs from a local symptomatic region (middle trapezius muscle, midpoint between the spinous process of C7 and the lateral border of the acromion) and a distant asymptomatic region (quadriceps muscle, midpoint between the anterior superior iliac spine and the basis patellae) [29]. For the measurement, pressure was gradually increased until the patients reported their first feeling of discomfort. PPTs were determined as the average of 2 consecutive measurements taken 30 s apart [29].

#### 2.2.6. Physical Capacity

The 2-Minute Walk Test (2-MWT) is a commonly used method for assessing overall physical capacity [48]. The 2-MWT was applied on a 15.2 m out-and-back course. Participants were instructed to walk as fast as they could without running until they were asked to stop. The distance walked by the participants was calculated and recorded in meters [48].

#### 2.2.7. Muscle Strength

A digital dynamometer (Kinvent Muscle Controller, New York, NY, USA) was used to evaluate the isometric strength of the cervical muscles. This is a commonly used method for the assessment of muscle strength [49]. In measuring cervical flexor muscle strength, the participant lay supine and was asked to resist pressure applied to the forehead during neck flexion. For the assessment of cervical extensor muscle strength, the participant lay prone with their neck extended and was asked to maintain this neck position against resistance applied to the occipital region. Cervical lateral flexor muscle strength was measured in a side-lying position, the neck was laterally flexed, and the participant was asked to maintain this position against resistance applied from the side of the head [50]. Each measurement was performed three times, and the average value was recorded in Newtons [49].

#### 2.2.8. Pain Catastrophizing Scale (PCS)

This scale was used to assess the level of pain catastrophizing [51]. The scale consists of 13 questions, and each question is scored between 0 and 4 points. The total score that can be obtained from the scale is between 0 and 52 points, with a higher score indicating more severe catastrophizing [52].

#### 2.2.9. Tampa Kinesiophobia Scale (TKS)

The TKS was administered to assess fear of movement and fear of reinjury [53]. The scale consists of 17 questions that identify movement-related fears, and each question is scored on a 4-point Likert scale (0–4), with a maximum total score of 68. A higher score indicates more severe kinesiophobia [54].

#### 2.2.10. Central Sensitization Inventory (CSI)

The CSI is a self-reported questionnaire used for assessing central sensitization [55]. The scale consists of 2 sections. Section A consists of 25 questions that inquire about the frequency of symptoms that may be associated with central sensitization, while Section B inquires whether the patient has been diagnosed (by a physician) with a disease thought to be associated with central sensitization. Only section A was used for assessment in this study [56]. Each question is scored from never (0) to always (4). Higher scores for this scale indicate that self-perceived central sensitization is more severe [57].

#### 2.2.11. Neuropsychological Assessment

The Standardized Mini-Mental State Test (SMMT), Trail Making Test (TMT), and Stroop Color Word Test (SCWT) were administered to the participants during the neuropsychological assessment [29,58,59]. The SMMT was used to determine the global cognition of participants. This test consists of 5 sections: orientation, registration memory, attention and calculation, recall, and language. Scores for this test can range from 0 to 30 points [60]. TMT and SCWT were administered to determine the attention and executive functioning of the participants. TMT comprises two parts: part A (TMT-A) assesses processing speed, and part B (TMT-B) assesses attention and executive functions [58,61,62]. Part A involves randomly arranged numbers from 1 to 25 within circles, and the participant is asked to connect the numbers. Part B involves randomly arranged numbers from 1 to 13 and letters from A to I within circles. The SCWT consists of five parts: part I involves reading color names printed in black, and part II assesses reading color names printed in different colors. In part III, the individuals are asked to tell the color of the colored printed circles, while in part IV, the task is to recognize the color of the printed neutral words. Finally, part V focuses on the ability to inhibit the automatic reading of color names printed in incongruent colors [58,59]. TMT-B minus TMT-A (TMT B-A) and the difference between SCWT parts V and III (SCWT V-III) completion times were also calculated in order to assess the attention, response inhibition, and cognitive flexibility of the participants.

### 2.3. Statistical Analyses

All data were analyzed using the Statistical Package for the Social Sciences (SPSS), version 23.0 (IBM Corp, Armonk, NY, USA). Distribution normality was investigated using visual (histograms and probability plots) and analytical methods (Shapiro–Wilk’s test). Variables with a normal distribution were presented as mean ± standard deviation (SD), non-normally distributed variables as median and interquartile ranges (IQR), and categorical variables as numbers (percentage). An independent sample *t*-test was performed to compare the SMMT total score according to gender, and the Kruskal–Wallis test was performed to compare the SMMT total score according to education level. The Spearman correlation coefficient test was used to assess the correlations between the parameters. A correlation coefficient between 0.00 and 0.10 was interpreted as representing negligible correlation, between 0.10 and 0.39 as weak correlation, between 0.40 and 0.69 as moderate correlation, between 0.70 and 0.89 as strong correlation, and between 0.90 and 1.00 as very strong correlation [63]. The magnitudes of the coefficients were taken into consideration when interpreting the correlation results in this study. A multiple linear regression analysis was performed to investigate the extent to which the independent variables were associated with cognitive performance. The assumptions of the multiple linear regression model were examined using the variance inflation factor (VIF) for multicollinearity, histograms and normal probability plots for the normality of the residuals, and by checking whether outliers/influential observations fell within the ±3 range of the standardized residuals and whether the Cook’s distance values of the observations were below 1. Additionally, the linearity and homoscedasticity of the model were examined by plotting scatter plots of the residuals against the estimated values. The significance level for all statistical analyses was set at *p* < 0.05.

Assuming an R^2^ value of 0.20 among the variables and a Cohen’s f effect size of $\frac{R^{2}}{1-R^{2}}$ = 0.25, the minimum required sample size was calculated as 69 for a model including eight independent variables, with a significance level of 0.05 and a statistical power of 0.80. On the other hand, according to the recommendation of at least 10 observations per variable in regression analysis [64], a minimum of 80 observations was deemed necessary.

## 3. Results

Of the 96 individuals referred to this study, 87 were included. Nine individuals were excluded due to not being willing to participate in the study (*n* = 5), having a neurological disease (*n* = 3), and illiteracy (*n* = 1). The median age of the participants was 49 (37–58) years. Most of the participants were female (65.50%), were right-handed (89.65%), worked in professional specialty occupations (35.60%), and were university graduates (37.90%). The demographic characteristics of the participants are presented in Table 1.

As shown in Table 2, according to NRS, the pain intensity of participants was 5.0 (2.0–7.0). The BMI of the participants was 26.19 ± 4.16 kg/m^2^. The 2-MWT distance was 184.24 (173.96–198.41) meters. The PPTs of the participants were 12.37 ± 5.77 lbf for the symptomatic region and 14.82 ± 5.12 lbf for the distal asymptomatic region. The mean of the SMMT total score was 27.49 ± 2.07 for female participants and 27.70 ± 1.57 for male participants; there was no statistically significant difference in SMMT total score according to gender (*p* > 0.05). In addition, participants with primary or secondary education had a lower total SMMT score than participants with postgraduate education (*p* = 0.001). The completion times for TMT-A and TMT-B were 37.00 (29.00–47.00) and 87.00 (51.00–137.00) seconds, respectively. Moreover, the SCWT part V completion time was 24.00 (19.00–32.00) seconds. The difference between the SCWT parts V and III completion times was 12.00 (7.00–18.00) seconds. The cervical muscle strengths of the participants were as follows: 50.39 ± 20.46 Newton (N) for cervical flexors, 64.69 ± 23.79 N for cervical extensors, 55.82 ± 21.44 N for cervical left lateral flexors, and 57.02 ± 22.35 N for cervical right lateral flexors. Detailed information regarding the clinical characteristics of the participants is presented in Table 2 and Table S1 (for SCWT I–IV).

The relationship between physical performance and pain-related parameters is presented in Table 3. The severity of pain catastrophizing was strongly related to neck disability level (r = 0.732); moderately related to cervical left lateral flexor muscle strength (r = −0.540), cervical flexor muscle strength (r = −0.501), pain intensity (r = 0.468), cervical right lateral flexor (r = −0.441) muscle strength, physical capacity (r = −0.421), and local (r = −0.411) and distal (r = −0.416) pain sensitivity; and weakly related to cervical extensor muscle strength (r = −0.397) in people with chronic neck pain (*p* < 0.001).

The severity of kinesiophobia was weakly correlated with pain intensity (r = 0.350), neck disability level (r = 0.345), physical capacity (r = −0.340), cervical left lateral flexor (r = −0.313), right lateral flexor (r = −0.230), and flexor muscle strength (r = −0.212) (*p* < 0.05). In contrast, there was no correlation between local and distal pain sensitivity, the muscle strength of cervical extensors, and the severity of kinesiophobia (*p* > 0.05).

Self-reported central sensitization severity was moderately associated with neck disability level (r = 0.673), cervical left lateral flexor (r = −0.531), right lateral flexor (r = −0.455), flexor muscle strength (r = −0.437), distal pain sensitivity (r = −0.414), cervical extensor muscle strength (r = −0.414), and physical capacity (r = −0.403), and weakly associated with pain intensity (r = 0.354) and local pain sensitivity (r = −0.333) (*p* < 0.05).

The relationship between cognitive performance, physical performance, and pain-related parameters is presented in Table 4.

According to the correlation analysis, the SMMT total score was moderately correlated with pain intensity (r = −0.427) and pain catastrophizing (r = −0.414) and weakly correlated with cervical extensor (r = 0.384), left lateral flexor (r = 0.380) muscle strength, age (r = −0.360), local pain sensitivity (r = 0.354), neck disability level (r = −0.353), cervical right lateral flexor (r = 0.336) muscle strength, physical capacity (r = 0.332), kinesiophobia (r = −0.313), distal pain sensitivity (r = 0.298), self-reported central sensitization (r = −0.241), and cervical flexor muscle strength (r = 0.233) in individuals with chronic neck pain (*p* < 0.05).

TMT-A completion time was moderately correlated with age (r = 0.487) and weakly correlated with kinesiophobia level (r = 0.328) and cervical extensor muscle strength (r = −0.222) (*p* < 0.05). Additionally, TMT-B completion time (r = 0.456) and TMT B-A (r = 0.407) were moderately correlated with age (*p* < 0.001). However, increased TMT-B completion time (r = 0.213) and TMT B-A (r = 0.218) demonstrated weak correlation with local pain sensitivity (*p* = 0.048) (*p* < 0.05). No significant associations were observed between other parameters and TMT completion time and TMT B-A (*p* > 0.05).

SCWT part V was weakly correlated with age (r = 0.357), physical capacity (r = −0.326), and pain intensity (r = 0.296) (*p* < 0.05). The difference between the completion times of SCWT parts V and III was moderately correlated with age (r = 0.440) and weakly correlated with physical capacity (r = −0.287) and pain intensity (r = 0.274) (*p* < 0.05). Nevertheless, neck disability level, local and distal pain sensitivity, cervical muscle strength, pain catastrophizing, kinesiophobia, and self-reported central sensitization were not correlated with SCWT completion time or SCWT V-III (*p* > 0.05). The relationship between SCWT I–IV completion time, physical performance, and pain-related psychosocial parameters is presented in Table S2.

A multiple linear regression analysis was performed to identify factors associated with global cognitive performance, attention, and executive functions. Detailed information regarding linear regression analysis is presented in Table 5. Age, pain intensity, physical capacity, PPT, muscle strength, neck disability level, kinesiophobia, and pain catastrophizing were included in the model. A stepwise selection method was applied. The normality of the residuals was evaluated using a histogram and a normal probability plot; all residuals were found to be consistent with a normal distribution. When multicollinearity among the independent variables was assessed using VIF, no values above 5 were observed in any of the models. When outliers and influential observations were examined using standardized residuals and Cook’s distance, only two observations in SCWT V and three observations in SCWT V-III had standardized residual values greater than 3; however, Cook’s distance values were less than 1 in all models.

For the SMMT total score, age, pain intensity, and PPT remained significant in the model. Age and pain intensity were negatively associated with global cognition, with decreases of 0.047 and 0.209 points in the total SMMT score, respectively. In contrast, PPT was positively associated with the SMMT total score, corresponding to an increase of 0.484 points in the total SMMT score. Among all variables, age exhibited the strongest association with the SMMT total score (β = −0.332), followed by pain intensity (β = −0.291) and PPT (β = 0.253). This model accounted for 30.8% of the variance in the SMMT total score.

Age, neck disability level, and kinesiophobia remained in the model for TMT-A completion time. These parameters were positively associated with the TMT-A completion time, with increases of 0.619, 0.730, and 1.042 s in completion time, respectively. Among these variables, age exhibited the strongest association with TMT-A completion time (β = 0.448), followed by kinesiophobia (β = 0.325) and neck disability level (β = 0.262). This model explained 34.2% of the variance in TMT-A completion time.

In addition, age and PPT showed a positive association with TMT-B completion time, with increases of 1.83 and 13.443 s in completion time, respectively. Age exhibited the strongest association (β = 0.430) with TMT-B completion time. The model accounted for 28.7% of the variance in TMT-B completion time.

Pain intensity was positively associated with TMT B-A, with an increase of 10.712 units in TMT B-A (β = 0.229) and an explained variance of 4.1%.

Age and pain intensity were positively associated with SCWT V completion time, with increases of 0.267 and 1.425 s in completion time, respectively. Among all variables, pain intensity exhibited the strongest association with SCWT V completion time (β = 0.322), followed by age (β = 0.309) and PPT (β = 0.178). This model accounted for 18.9% of the variance in SCWT V completion time.

Additionally, kinesiophobia was associated with SCWT V-III, with an increase of 0.331 units in SCWT V-III (β = 0.214) and an explained variance of 3.5%.

Variables associated with the SCWT I–IV in people with chronic neck pain are presented in Table S3.

## 4. Discussion

The results of this study add to the growing body of evidence suggesting that reduced physical performance is associated with demographic and pain-related psychosocial factors in individuals with chronic neck pain. In addition, pain-related physical and psychosocial impairments may be related to impairments across different cognitive domains in individuals with chronic neck pain. Furthermore, older age, higher pain intensity, decreased pressure pain thresholds, higher levels of neck disability, kinesiophobia, and decreased cervical muscle strength were associated with cognitive impairments across different cognitive domains in these individuals.

Chronic neck pain is a prevalent musculoskeletal condition that significantly affects physical function and psychological factors [22,28]. Beyond structural impairments, psychological parameters such as kinesiophobia, central sensitization, and pain catastrophizing play important roles in the persistence and severity of symptoms in individuals with chronic neck pain [28,65,66]. In line with previous studies, this study suggests clinically meaningful associations between pain-related psychosocial and physical parameters in people with chronic neck pain. Increased pain catastrophizing, kinesiophobia, and self-reported central sensitization were associated with increased pain intensity, neck disability level, lower physical capacity, and lower cervical muscle strength in people with chronic neck pain. Additionally, PPTs were associated with increased pain catastrophizing and self-reported central sensitization. These findings indicate that increased nociceptive sensitization and decline in physical performance may predispose individuals to maladaptive coping strategies related to pain. Decreases in physical capacity and muscle strength, as well as an increase in pain sensitivity, may exacerbate neck pain during daily living activities; therefore, people with chronic neck pain may experience higher levels of kinesiophobia, central sensitization, and pain catastrophizing. Because of this, it may be important to consider the relationship between pain-related psychosocial parameters and physical impairments in these individuals.

The association between pain intensity and cognitive performance has been addressed comprehensively in the literature, with consistent evidence indicating that individuals with chronic pain exhibit impairments across multiple cognitive domains [33,67,68]. Existing evidence suggests that individuals with chronic neck pain may experience clinically meaningful cognitive difficulties across different cognitive domains, including global cognition, attention, concentration, and executive functions [22,29,37,65,69]. In addition, demographic and clinical factors such as older age, higher pain intensity, central sensitization, pain catastrophizing, and neck disability level have been proposed as contributors to cognitive decline in the chronic neck pain population [22,36,37,69].

Consistent with prior evidence, this study suggests that cognitive impairment in individuals with chronic neck pain may not be a unitary phenomenon but rather a multifaceted consequence of overlapping physical, psychosocial, and sensory mechanisms, with distinct profiles emerging across different cognitive domains. Global cognition was independently associated with older age, higher pain intensity, and decreased pressure pain thresholds, together explaining approximately 31% of the variance. This pattern suggests that the relationship between nociceptive sensitization and aging-related neurobiological decline may be particularly detrimental to overall cognitive integrity [69,70]. Notably, processing speed was uniquely associated with neck disability level and kinesiophobia, independent of pain intensity itself. This domain-specific finding implies that functional disability and fear-avoidance behavior—rather than nociceptive input alone—may drive slowed information processing, possibly through sustained attentional capture by threat-related cognitions [71]. In a parallel vein, response inhibition was exclusively related to kinesiophobia, pointing to a specific role for movement-related fear in disrupting inhibitory control. Furthermore, cervical muscle strength independently correlated with color-naming speed, suggesting that peripheral neuromuscular capacity may partially contribute to basic perceptual processing efficiency, potentially via shared corticomotor and attentional circuits. These domain-specific associations may provide a more granular account of cognitive vulnerability in chronic neck pain and could set the stage for the possible associations elaborated below.

Muscle strength is an important marker for peripheral neuromuscular capacity and central nervous system integration. Impairment in peripheral neuromuscular capacity has been shown to correlate with cognitive decline in adults without chronic pain [72,73]. Decreased muscle strength may be related to decreased levels of neurotrophic factors in the central nervous system [74,75]. Additionally, lower physical capacity may also be associated with decreased cerebral perfusion [76], and this may be linked to a decrease in cognitive performance [77]. These possible changes in the central nervous system may be associated with a decrease in global cognition, particularly in attention and executive functions. To the best of our knowledge, this is the first study to identify the relationship between peripheral neuromuscular capacity, reduced physical capacity, and cognitive functioning in individuals with chronic neck pain. These findings may represent a novel contribution to the existing literature. Furthermore, increased pain intensity, central sensitization, pain catastrophizing, and kinesiophobia were consistently associated with worse cognitive outcomes, supporting the role of altered nociceptive processing in cognitive dysfunction [70]. We tentatively propose that the observed associations between pain-related parameters and cognitive performance may be interpreted within the framework of the triple network model [78], though we emphasize that this should be considered hypothesis-generating in the absence of direct neuroimaging evidence in this specific population. This model conceptualizes the dynamic interaction of the salience network (SN), the default mode network (DMN), and the central executive network (CEN) [78,79]. In chronic pain conditions, it has been hypothesized that increased SN activity and reduced deactivation of DMN may contribute to dysregulation of the CEN, which in turn may be associated with cognitive and behavioral difficulties [80]. Additionally, pain-related somatosensory activity may become functionally coupled with the DMN, suggesting that prolonged pain acts as an intrinsic part of the self-perception. This altered interaction between the DMN and frontoparietal CEN may be associated with cognitive and psychosocial difficulties in chronic pain populations [78]. From this hypothesis-generating perspective, prolonged pain and associated deterioration in physical and psychosocial parameters may be linked to altered SN and DMN activity, alongside reduced CEN engagement, which may ultimately be associated with poorer cognitive performance in individuals with chronic neck pain. Future neuroimaging studies are needed to test this framework directly in individuals with chronic neck pain.

### 4.1. Limitations

This study has several limitations. Parameters such as pain intensity, neck disability level, pain catastrophizing, kinesiophobia, and self-reported central sensitization are inherently subjective and rely on participants’ personal perception and experience, which may have limited the variability of the dependent variable and, consequently, the explanatory power of the regression model. Additionally, although participants with known neurological, metabolic, cardiovascular, and/or inflammatory systemic diseases were excluded, medication use beyond analgesics was not directly controlled in this study. In addition, although pain-related psychosocial parameters such as pain catastrophizing, kinesiophobia, and self-reported central sensitization were assessed in this study, other psychosocial parameters that could affect cognitive status, such as stress, anxiety, depression, or sleep disturbance, were not. It should be considered that factors such as sleep quality, medication use, and psychological distress (e.g., anxiety and depression) may also be associated with cognitive performance and may account for a substantial portion of the unexplained variance. Moreover, even if all the assumptions of linear regression were met, the use of stepwise regression may increase the risk of overfitting and Type I errors due to the data-driven variable selection process. Therefore, the identified variables should be interpreted with caution and validated in independent samples.

Although the relationship between decreased muscle strength, physical capacity, and cognitive performance has been addressed from a neurobiological perspective, this study did not include additional examinations such as neuroimaging and biomarkers, which could have provided more comprehensive and objective assessments. Therefore, it should be kept in mind that these relationships are hypothetical. Despite all assessment tools used in this study have established validity and reliability in general populations, they have not been specifically validated (except the NDI) in individuals with chronic neck pain. Therefore, the findings should be interpreted with caution, particularly for measures that may be influenced by pain-related factors such as variability in performance or altered pain perception. Also, only the neck flexor, extensor, and lateral flexor muscles were examined in this study. It may be useful to also evaluate rotational forces in future studies.

Additionally, as participants were recruited from a single center, the generalizability of the findings may be limited. Finally, causal relationships between variables cannot be established due to the cross-sectional study design.

Despite the limitations mentioned above, to the best of our knowledge, this is the first study to investigate the associations between kinesiophobia, physical capacity, cervical muscle strength, and cognitive performance in people with chronic neck pain. In addition, this study contributes to the literature and may have implications for clinical practice regarding pain-related impairments in physical, psychosocial, and cognitive performance and their interrelationships in this population.

### 4.2. Implications for Clinical Practice

The findings of this study may guide clinicians in identifying important parameters that should be taken into account during the assessment of and treatment planning for people with chronic neck pain. Although the cross-sectional design prevents causal inference, the observed associations in this study suggested that, in addition to assessing physical and psychosocial parameters, examination of cognitive performance may also be an important part of clinical assessments of these individuals. In particular, patients presenting with older age, higher pain intensity, elevated kinesiophobia, or reduced cervical muscle strength may represent a priority subgroup for integrated cognitive screening, as these factors were independently associated with cognitive deficits in the regression analyses. The findings of our study suggested that, in order to provide personalized treatment targeting the impairments observed in this population, physical impairments, psychosocial conditions, cognitive impairments, and the relationships among them should be assessed. These findings also suggest that it may be beneficial to incorporate biopsychosocial approaches—such as cognitive behavioral therapy or pain neuroscience education—alongside traditional strategies focused on pain management and improving physical performance to modify maladaptive behaviors related to pain and support improvements in cognitive functioning by decreasing the cognitive load and attentional burden associated with chronic pain in these individuals.

## 5. Conclusions

The present study aimed to examine the relationship between physical functions, psychosocial status, and cognitive performance in individuals with chronic neck pain and explore the factors influencing cognitive performance in these people. These findings suggest that higher pain intensity, increased neck disability level, lower physical capacity, and reduced cervical muscle strength may be associated with increased pain catastrophizing, kinesiophobia, and self-reported central sensitization in people with chronic neck pain. In addition, we observed a correlation between higher levels of pain catastrophizing, self-reported central sensitization, and increased pain sensitivity in these people. Moreover, older age, higher pain intensity, lower physical capacity, increased pain sensitivity, reduced cervical muscle strength, greater neck disability, higher pain catastrophizing, greater kinesiophobia, and increased self-reported central sensitization may also be associated with cognitive deficits. Furthermore, older age, higher pain intensity, and increased pain sensitivity may be negatively related to global cognition in these people. Age, functional disability, and kinesiophobia were correlated with the individual’s processing speed. Finally, attention and executive functioning may be related to age, pain intensity, cervical muscle strength, pain sensitivity, neck disability level, and kinesiophobia. These findings contribute to the growing body of evidence suggesting that physical and psychosocial impairments may be associated with cognitive performance in individuals with chronic neck pain. Future studies should focus not only on physical and psychosocial parameters but also on cognitive parameters. Investigating the neurobiological mechanisms underlying these cognitive changes may be important.

## Figures and Tables

**Table 1:** Demographic characteristics of the participants.

Variables	Participants (*n* = 87)
	**Median (IQR 25–75)**
Age (years)	49 (37–58)
	***n*** **(%)**
Gender	
Female	57 (65.50)
Male	30 (34.50)
Hand Dominance	
Right-handed	78 (89.65)
Left-handed	9 (10.35)
Ambidextrous	0 (0)
Job Score	
I	18 (20.70)
II	11 (12.60)
III	7 (8.00)
IV	10 (11.50)
V	10 (11.50)
VI	31 (35.60)
Education Level	
Primary school	12 (13.80)
Secondary school	4 (4.60)
High school	21 (24.10)
University	33 (37.90)
Postgraduate	17 (19.50)

**Table 2:** Clinical characteristics of the participants.

Clinical Aspects	Participants (*n* = 87)
	**Median (IQR 25–75)**
Pain intensity	5.0 (2.0–7.0)
2-MWT (m)	184.24 (173.96–198.41)
NDI (scores)	9.00 (6.00–13.00)
CSI (scores)	31.00 (22.00–45.00)
PCS (scores)	12.00 (6.00–28.00)
Cognitive Tests	
SMMT (score)	28.00 (26.00–29.00)
TMT-A (s)	37.00 (29.00–47.00)
TMT-B (s)	87.00 (51.00–137.00)
SCWT (s)	
Part V	24.00 (19.00–32.00)
Part V–III	12.00 (7.00–18.00)
	**Mean ± SD**
BMI (kg/m^2^)	26.19 ± 4.16
PPT (lbf)	
LSR	12.37 ± 5.77
DAR	14.82 ± 5.12
TKS (scores)	36.80 ± 6.10
Muscle Strength (N)	
Cervical flexors	50.39 ± 20.46
Cervical extensors	64.69 ± 23.79
Cervical lateral flexors (L)	55.82 ± 21.44
Cervical lateral flexors (R)	57.02 ± 22.35

Note: 2-MWT: Two-Minute Walk Test; CSI: Central Sensitization Inventory; DAR: distal asymptomatic region; L: left; LSR: local symptomatic region; NDI: Neck Disability Index; PCS: Pain Catastrophizing Scale; PPT: pressure pain threshold; R: right; SMMT: Standardized Mini-Mental Test; SCWT: Stroop Color Word Test; TKS: Tampa Kinesiophobia Scale; TMT-A: Trail Making Test Part A; TMT-B: Trail Making Test Part B.

**Table 3:** Correlations between physical performance and psychosocial parameters.

Measure		PCS	TKS	CSI
Pain intensity	rho	0.468 **	0.350 **	0.354 **
	*p*	<0.001	0.001	0.001
NDI	rho	0.732 **	0.345 **	0.673 **
	*p*	<0.001	0.001	<0.001
2-MWT	rho	−0.421 **	−0.340 **	−0.403 **
	*p*	<0.001	0.001	<0.001
PPT				
LSR	rho	−0.411 **	−0.185	−0.333 **
	*p*	<0.001	0.086	0.002
DAR	rho	−0.416 **	−0.176	−0.414 **
	*p*	<0.001	0.104	<0.001
Muscle Strength				
Cervical flexors	rho	−0.501 **	−0.212 *	−0.437 **
	*p*	<0.001	0.048	<0.001
Cervical extensors	rho	−0.397 **	−0.185	−0.404 **
	*p*	<0.001	0.087	<0.001
Cervical lateral flexors (L)	rho	−0.540 **	−0.313 **	−0.531 **
	*p*	<0.001	0.003	<0.001
Cervical lateral flexors (R)	rho	−0.441 **	−0.230 *	−0.455 **
	*p*	<0.001	0.032	<0.001

Note: 2-MWT: Two-Minute Walk Test; DAR: distal asymptomatic region; L: left; LSR: local symptomatic region; PPT: pressure pain threshold; R: right; rho: Spearman’s correlation coefficient; * Correlation is significant at the 0.05 level (2-tailed); ** Correlation is significant at the 0.01 level (2-tailed).

**Table 4:** Correlations between cognitive performance, age, physical performance, and pain-related parameters.

Measure		SMMT	TMT A	TMT B	TMT B-A	SCWT V	SCWT V-III
Age	rho	−0.360 **	0.487 **	0.456 **	0.407 **	0.357 **	0.440 **
	*p*	<0.001	<0.001	<0.001	<0.001	0.001	<0.001
Pain intensity	rho	−0.427 **	0.130	0.096	0.138	0.296 **	0.274 *
	*p*	<0.001	0.229	0.377	0.203	0.005	0.010
NDI	rho	−0.353 **	0.171	0.042	0.065	0.130	0.141
	*p*	0.001	0.114	0.699	0.549	0.232	0.192
2-MWT	rho	0.332 **	−0.200	−0.153	−0.146	−0.326 **	−0.287 **
	*p*	0.002	0.064	0.156	0.178	0.002	0.007
PPT							
LSR	rho	0.354 **	0.125	0.213 *	0.218 *	0.096	0.122
	*p*	0.001	0.248	0.048	0.043	0.376	0.260
DAR	rho	0.298 **	0.027	0.182	0.157	0.034	0.029
	*p*	0.005	0.804	0.091	0.147	0.756	0.789
Muscle Strength							
Cervical flexors	rho	0.233 *	0.041	0.118	−0.023	−0.123	−0.107
	*p*	0.030	0.708	0.276	0.832	0.256	0.322
Cervical extensor	rho	0.384 **	−0.222 *	−0.090	−0.077	−0.167	−0.157
	*p*	<0.001	0.039	0.407	0.477	0.122	0.147
Cervical lateral flexor (L)	rho	0.380 **	−0.144	−0.062	−0.166	−0.150	−0.135
	*p*	<0.001	0.182	0.568	0.125	0.164	0.213
Cervical lateral flexor (R)	rho	0.336 **	−0.141	−0.105	−0.186	−0.196	−0.137
	*p*	0.001	0.191	0.333	0.084	0.069	0.207
PCS	rho	−0.414 **	0.199	−0.012	−0.049	0.103	0.093
	*p*	<0.001	0.065	0.909	0.651	0.344	0.389
TKS	rho	−0.313 **	0.328 **	0.138	0.086	0.204	0.172
	*p*	0.003	0.002	0.202	0.429	0.059	0.111
CSI	rho	−0.241 *	−0.009	−0.158	−0.143	0.029	−0.021
	*p*	0.024	0.932	0.145	0.186	0.787	0.848

Note: 2-MWT: Two-Minute Walk Test; CSI: Central Sensitization Inventory; DAR: distal asymptomatic region; L: left; LSR: local symptomatic region; NDI: Neck Disability Index; PCS: Pain Catastrophizing Scale; PPT: pressure pain threshold; R: right; SMMT: Standardized Mini-Mental Test; TKS: Tampa Kinesiophobia Scale; TMT-A: Trail Making Test Part A; TMT-B: Trail Making Test Part B. * Correlation is significant at the 0.05 level (2-tailed); ** Correlation is significant at the 0.01 level (2-tailed).

**Table 5:** Variables associated with cognitive performance in people with chronic neck pain.

Cognitive Test	Variable	B	SE	β	CI 95%		t	*p*	Tolerance	VIF	
SMMT	Age	−0.047	0.013	−0.332	−0.072	−0.0216	−3.650	<0.001	0.843	1.187	Adjusted R^2^ = 0.308 F = 13.750 *p* < 0.001
	Pain Intensity	−0.209	0.070	−0.291	−0.347	−0.071	−2.974	0.004	0.975	1.025	
	PPT	0.484	0.185	0.253	0.121	0.847	2.614	0.011	0.856	1.169	
TMT A	Age	0.619	0.123	0.448	0.378	0.860	5.034	<0.001	0.967	1.034	Adjusted R^2^ = 0.342 F = 12.912 *p* < 0.001
	NDI	0.730	0.331	0.262	0.082	1.378	2.207	0.030	0.543	1.843	
	TKS	1.042	0.314	0.325	0.426	1.658	3.314	0.001	0.797	1.255	
TMT B	Age	1.833	0.398	0.430	1.053	2.613	4.605	<0.001	0.949	1.054	Adjusted R^2^ = 0.287 F = 7.932 *p* < 0.001
	PPT	13.443	6.458	0.232	0.785	26.102	2.082	0.041	0.665	1.504	
	Muscle Strength	−13.110	6.808	−0.227	−26.454	0.234	−1.926	0.058	0.598	1.671	
	TKS	1.826	1.007	0.185	−0.148	3.800	1.813	0.074	0.800	1.251	
TMT B-A	Pain Intensity	10.712	4.934	0.229	1.040	20.383	2.171	0.033	1.000	1.000	Adjusted R^2^ = 0.041 F = 4.713 *p* = 0.033
SCWT Part V	Age	0.267	0.085	0.309	0.100	0.434	3.140	0.002	0.975	1.025	Adjusted R^2^ = 0.189 F = 7.694 *p* < 0.001
	Pain Intensity	1.425	0.468	0.322	0.507	2.342	3.043	0.003	0.843	1.187	
	PPT	2.087	1.234	0.178	−0.331	4.506	1.692	0.094	0.856	1.169	
SCWT V-III	TKS	0.331	0.164	0.214	0.009	0.652	2.021	0.046	1.000	1.000	Adjusted R^2^ = 0.035 F = 4.084 *p* = 0.046

Note: β: standardized coefficients; B: unstandardized coefficients; SCWT: Stroop Color Word Test; PPT: pressure pain threshold; TKS: Tampa Kinesiophobia Scale; SE: standard error; SMMT: Standardized Mini-Mental Test; VIF: variance inflation factor.

## Data Availability

The data presented in this study are available upon request from the corresponding author. Data are not publicly available due to ethical restrictions.

## Supplementary Materials

The following supporting information can be downloaded at https://www.mdpi.com/article/10.3390/medicina62050956/s1, Table S1. Participant SCWT I–IV completion time; Table S2. Correlations between SCWT I–IV, age, physical performance, and pain-related parameters; Table S3. Significant predictors of SCWT I–IV in people with chronic neck pain.

## Abbreviations

The following abbreviations are used in this manuscript:

NDI	Neck Disability Index
PPT	Pressure Pain Threshold
2-MWT	2-Minute Walk Test
PCS	Pain Catastrophizing Scale
TKS	Tampa Kinesiophobia Scale
CSI	Central Sensitization Inventory
SMMT	Standardized Mini-Mental State Test
TMT	Trail Making Test
SCWT	Stroop Color Word Test
SN	Salience Network
DMN	Default Mode Network
CEN	Central Executive Network
