# Supplementary material for: Multidimensional Analysis of Physical, Psychosocial, and Cognitive Impairment in People with Chronic Neck Pain
Source: Medicina (Kaunas). 2026 May 14;62(5):956. doi: 10.3390/medicina62050956 (PMC13208137; doi:10.3390/medicina62050956)
Supplement: Supplementary file 1 [file medicina-62-00956-s001.zip › medicina-4289697-supplementary.pdf]

Table S1. SCWT I-IV completion time of the participants

Clinical Aspects	Participants (n=87)
SCWT (sec)	
Part I	10.00 (8.00-12.00)
Part II	11.00 (9.00-13.00)
Part III	12.00 (11.00-16.00)
Part IV	17.00 (13.00-22.00)

Note: SCWT: Stroop Color Word Test

Table S2. Correlations between SCWT I-IV, age, physical performance, and pain-related parameters

Measure		SCWT I	SCWT II	SCWT III	SCWT IV
Age	rho	0.280**	0.294**	0.315**	0.487**
	p	0.009	0,006	0.003	<0.001
Pain intensity	rho	0.221*	0.234*	0.108	0.196
	p	0.039	0.029	0.319	0.069
NDI	rho	0.079	0.058	-0.083	-0.012
	p	0.467	0.595	0.443	0.913
2MWT	rho	-0.210	-0.222*	-0.192	-0.264*
	p	0.051	0.039	0.074	0.013
PPT					
LSR	rho	-0.024	-0.228*	0.193	0.059

	p	0.824	0.033	0.073	0.584
DAR	rho	-0.052	-0.229*	0.154	-0.003
	p	0.633	0.033	0.153	0.978
Muscle Strength					
Cervical flexors	rho	-0.232*	-0.130	-0.043	0.001
	p	0.030	0.230	0.693	0.996
Cervical extensor	rho	-0.268*	-0.278**	-0.113	-0.201
	p	0.012	0.009	0.298	0.062
Cervical lateral flexor (L)	rho	-0.245*	-0.248*	-0.096	-0.149
	p	0.022	0.020	0.374	0.168
Cervical lateral flexor (R)	rho	-0.204	-0.199	-0.096	-0.158
	p	0.058	0.065	0.377	0.144
PCS	rho	0.101	0.168	-0.003	0.013
	p	0.352	0.120	0.981	0.904
TKS	rho	0.273*	0.260*	0.112	0.199
	p	0.011	0.015	0.303	0.064
CSI	rho	0.109	0.110	-0.025	-0.059
	p	0.314	0.312	0.816	0.590

Note: 2-MWT: Two-Minute Walk Test; CSI: Central Sensitization Inventory; DAR: distal asymptomatic region; L: left; LSR: local symptomatic region; NDI: Neck Disability Index; PCS: Pain Catastrophizing Scale; PPT: pressure pain threshold; R: right; TKS: Tampa Kinesiophobia Scale; * Correlation is significant at the 0.05 level (2-tailed); ** Correlation is significant at the 0.01 level (2-tailed).

Table S3. Variables associated with the SCWT I-IV in people with chronic neck pain

Prediction	Variable	B	SE	β	CI95%		t	p	Tolerance	VIF	
**Stroop I**	**Age**	0.039	0.017	0.228	0.005	0.0733	2.232	0.028	0.967	1.034	Adjusted R^2=0.132 F=5.361 p=0.002
	**Muscle Strength**	-0.430	0.242	-0.185	-0.905	0.0447	-1.775	0.080	0.930	1.076	
	**TKS**	0.074	0.042	0.186	-0.008	0.156	1.774	0.080	0.913	1.095	
**Stroop II**	**Age**	0.089	0.030	0.303	0.031	0.148	3.013	0.003	0.999	1.001	Adjusted R^2=0.131 F=7.508 p=0.001
	**PPT**	-1.025	0.403	-0.256	-1.815	-0.235	-2.543	0.013	0.999	1.001	
**Stroop III**	**Age**	0.088	0.030	0.292	0.029	0.147	2.931	0.004	0.972	1.029	Adjusted R^2=0.170 F=5.397 p=0.001
	**PPT**	1.287	0.485	0.314	0.336	2.238	2.652	0.010	0.688	1.454	
	**Muscle Strength**	-1.582	0.575	-0.386	-2.709	-0.456	-2.753	0.007	0.490	2.039	
**Stroop IV**	**Age**	0.244	0.049	0.469	0.149	0.339	5.025	<0.001	0.966	1.035	Adjusted R^2=0.276 F=9.206 p<0.001
	**Muscle Strength**	-1.285	0.750	-0.182	-2.755	0.184	-1.714	0.090	0.748	1.337	
	**TKS**	0.239	0.124	0.198	-0.003	0.482	1.930	0.057	0.800	1.250	

Note: β: standardized coefficients; B: unstandardized coefficients; PPT: pressure pain thresh-old; TKS: Tampa Kinesiophobia Scale; SE: standard error; VIF: variance inflation factor; * p<0.05.
